# Is Resveratrol a Cancer Immunomodulatory Molecule?

**DOI:** 10.3389/fphar.2018.01255

**Published:** 2018-11-06

**Authors:** Ly Quoc Trung, Dao T. T. An

**Affiliations:** ^1^Soctrang Provincial Health Department, Soc Trang, Vietnam; ^2^Phuong Chau International Hospital, Can Tho, Vietnam; ^3^Pediatric Department, University of Medicine and Pharmacy, Ho Chi Minh City, Vietnam

**Keywords:** resveratrol, anti cancer, immunotherapy, NKG2D, melinjo

Immunomodulation is a component of immunotherapy in which immune responses are induced, amplified, attenuated, or prevented according to therapeutic goals. In the field of oncology this imply the administration of synthetic or natural agents capable of modulating patients' immune responses aimed to eradicate, arrest or prevent the dissemination of cancer cells. The achievement of this goal is clearly exemplified with the therapeutic efficacy of immune check-point inhibitors (Alsaab et al., 2017) whose application in the clinical setting has demonstrated not only that the immune system can be harnessed to fight cancer but also that immunotherapy can be incorporated into the anticancer arsenal.

In addition, the possibility to enhance endogenous immune functions with the use of vitamins and micronutrients, and more recently with the use of nutraceuticals (pharmaceutical-grade and standardized nutrients), has also been investigated. Several nutraceuticals have been reported to modulate in immune functions, most of them tested in preclinical studies (Nasri et al., 2014), however whether those substances, indeed act as immune boosters and are useful to improve immune function has not been systematically investigated in well-controlled human studies. One of the best studied nutraceuticals is resveratrol, which is a small polyphenol that was first isolated in 1940 from the roots of white *Veratrum grandiflorum* and later isolated from grapes, wine, berries, and other natural sources (Varoni et al., 2016). This compound has attracted considerable attention for its reported anti-cancer, antioxidant and anti-inflammatory properties with potential utility for the prevention or treatment of cardiovascular diseases, autoimmune disorders, neuro degenerative diseases, and other chronic disorders (Berman et al., 2017). Numerous studies using several type of tumor cells and distinct preclinical tumor models have shown that resveratrol has direct anti-tumor properties by modulating multiple signal pathways that are dysregulated in cancer (Quoc Trung et al., 2013; Gambini et al., 2015; Trung et al., 2015). Nevertheless, the efficacy of resveratrol as an anticancer agent is far to be proven since it has not been formally tested in human studies. In addition, it must be noted that in many studies reporting the anticancer properties of resveratrol, this compound was utilized at extremely high concentrations that are unlikely to be achieved *in vivo* given the poor bioavailability (<1%) of orally administered resveratrol, consequence of the extensive metabolism of this molecule in the liver and intestines (Walle, 2011). Notably, despite its poor bioavailability small clinical studies have documented that indeed orally given resveratrol can reach malignant tissues in patients with cancer (especially digestive tumors) and is capable of inducing moderate tumor inhibitory effects (Patel et al., 2010).

An area of growing interest is the reported ability of resveratrol to induce immunomodulatory effects with potential antitumor utility, which appears to be mediated by directly acting on immune cells or indirectly by sensitizing tumor cells to the cytotoxic effects of immune cells.

## Indirect immunomodulatory properties of resveratrol

Evidences of the indirect effects of resveratrol on immune system comes from *in vitro* studies, in which resveratrol was reported to induce or enhance the expression of stress markers on cancer cells making them more sensitive to immune cells attack. Early studies indeed reported that, by inducing the expression of death proteins on the surface of tumor cells, including the death receptor 4 and death receptor 5 (DR4 and DR5 respectively), resveratrol was able to improve the tumor cells elimination by immune cells, especially natural killer cells (NK cells) expressing the corresponding TNF-related apoptosis-inducing ligand (TRAIL) (Hu et al., 2012; Pan et al., 2017). In addition, several groups have independently shown the induction of stress ligands of NKG2D (including the MICA/B and ULBP ligands) on the surface of various tumor cell types and the corresponding engagement by immune cells expressing the activator receptor NKG2D, such as NK cells and γδT-lymphocytes ultimately results in the elimination of tumor cells expressing those stress ligands (Hu et al., 2012; Luis Espinoza et al., 2013; Pan et al., 2017). It is still unknown if the administration of resveratrol in patients with cancer may indeed lead to the expression of NKG2D ligands in tumor cells *in vivo*.

## Direct immunomodulatory properties of resveratrol

On the other hand, other studies have shown that resveratrol can affect immune cells directly. For example, resveratrol has been shown to promote the release of various proinflammatory cytokines from immune cells, which ultimately promotes cytotoxicity against cancer (Bergman et al., 2013; Schwager et al., 2017; Smith et al., 2018). Studies using immunodeficient mice reported the recovery of certain immune functions of animals receiving resveratrol, including an increase in splenocytes proliferation, larger number of circulating CD4+ cells and increased serum levels of cytokines in resveratrol-treated mice (Lai et al., 2016, 2017), although it is unknown if those changes can be indeed translated into enhanced immune functions since no functional studies (for example, the clearance of tumor cells or infected cells) were included in those studies (Lai et al., 2016, 2017).

Recently, in a xerograph model of human papilloma virus (HPV+) tumor, resveratrol given in combination with other polyphenols, enhanced tumor rejection by modulating immune functions of macrophages and by recruiting NK cells to the sites of tumor via IL-12 release (Mukherjee et al., 2018). However, a recent study showed that various colorectal and breast cancer lines exposed to high concentrations of resveratrol upregulated the programed cell death ligand 1 (PD-L1), a molecule that promotes tumor evasion of immune responses by interacting with the death receptor PD-1 expressed on T-cells (Lucas et al., 2018). Whether this phenomenon can be recapitulated *in vivo* is unknown and is an attractive line of investigation for future studies. For example, a potential upregulation of PD-L1 *in vivo* induced by resveratrol would render tumors more sensitive to immune check point inhibitors targeting PD-L1, however this phenomenon may also raise the concern that in individuals without cancer, the prolonged administration of resveratrol could favor cancer development since a putative upregulation of PD-L1 by resveratrol in transformed cells could promote immune evasion and tumor development. Further studies are needed to clarify this issue, especially for the fact that the concentrations of resveratrol used in the experiments reporting PD-L1 upregulation in tumor cells were very high (Lucas et al., 2018).

## Immunomodulatory properties of resveratrol: the *in vivo* evidence.

**Figure 1:**
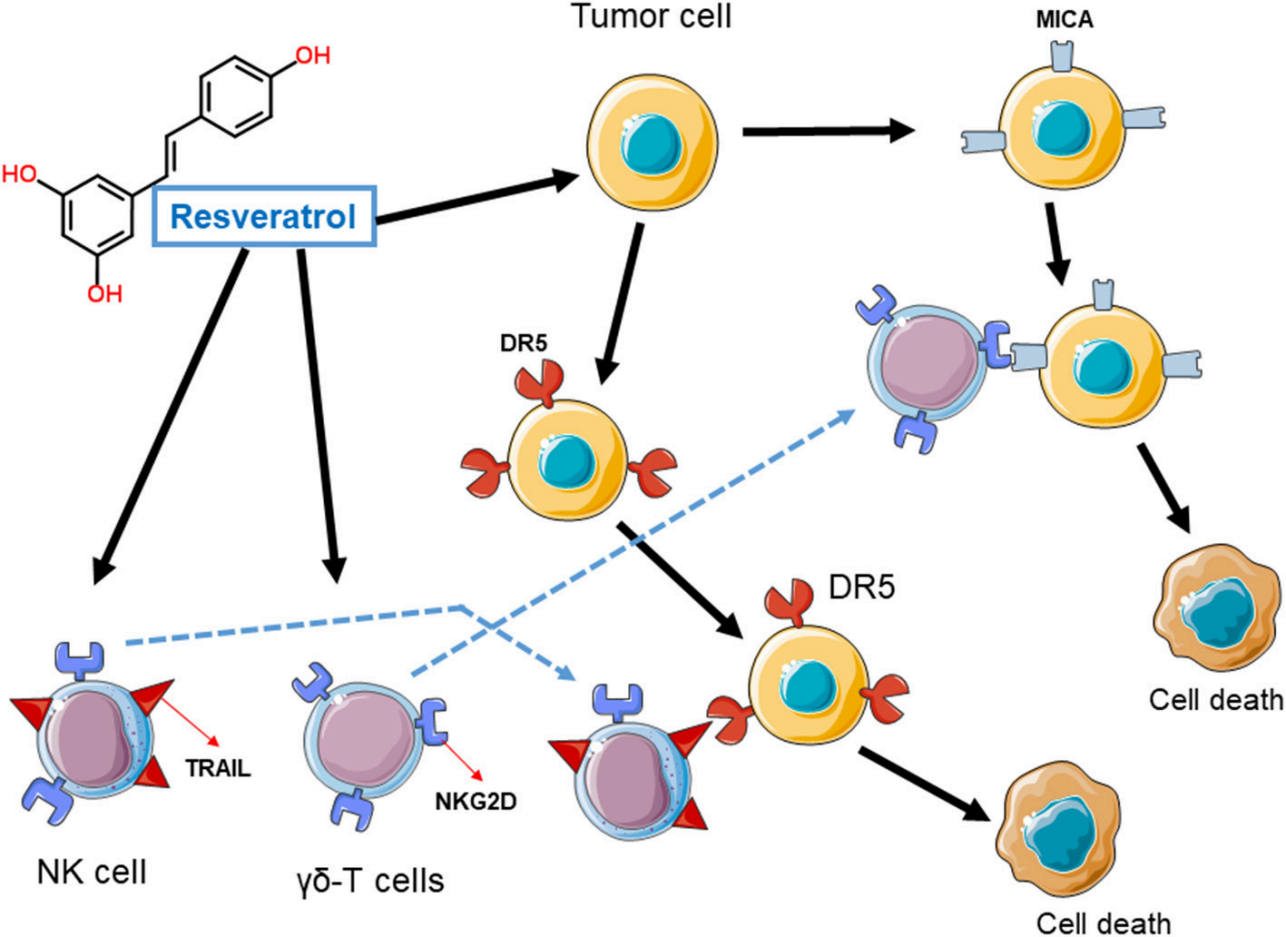
Induction of stress ligands in tumor cells by resveratrol. Cancer cells exposed to resveratrol upregulate stress proteins, such as death receptor 5 (DR5) or ligands for the immune receptor NKG2D including MICA or ULBPs. The engagement of these proteins by immune cells expressing the corresponding receptor (NKG2D receptor) or ligand (TRAIL), result in cancer cell death.

The consumption of resveratrol for up to 4 weeks in healthy individuals revealed directly modulatory effects on immune cells, including an increase in the number of circulating γδT-lymphocytes expressing NKG2D receptor in healthy individuals (Espinoza et al., 2017) and thus, considering that γδT-cells possess robust antitumor properties and are implicated in cancer immunosurveillance (Kabelitz and Déchanet-Merville, 2015) it is expected that resveratrol may prevent tumorigenesis in humans by enhancing immunosurveillance, although more studies are needed to support this assumption (Figure 1). In addition, a highly active nano-formulation of resveratrol was reported to increased expression of pluripotency transcripts on circulating blood cells (Oct-4A, Nanog and Sox2) and anti-aging and tumor suppressor transcripts NAD, SIRT1, SIRT6, and p53 in circulating progenitors cells in the blood of healthy individuals (Tripathi et al., 2017). Furthermore, a comprehensive analysis of publicly available human microarray data for significant gene expression changes associated with dietary intervention with EPA/DHA, flavonoids and resveratrol revealed an enrichment of genes implicated in immune responses and disease pathways which was common to all of the treatment conditions tested (Warburton et al., 2018). The above studies indicate that resveratrol clearly exert measurable effects in human immune cells, however, further studies are needed to clarify the reported immunomodulatory properties of this polyphenol. In special, well-designed double blinded randomized trials to test the effects of resveratrol in a large number of individuals are needed and these studies should include appropriate surrogate biomarkers indicative of immune response such as serum cytokine levels, immune cell subsets or gene expression signature. Another important issue to consider is the selection of the optimal target population for clinical trials of resveratrol since the immune responses associated with resveratrol intake may be different in healthy individuals than in high-risk populations, such as in heavy-smokers, elderly individuals or in people with precancerous lesions. Another important factor to consider would be the selection of the optimal dose of resveratrol. Although several studies have demonstrated that resveratrol given at high doses is safe for humans (Brown et al., 2010; Erdogan and Vang, 2016; Luis Espinoza et al., 2017), selecting a high dose is not necessarily the best strategy to achieve the goals since dietary low level doses of resveratrol may indeed exert biological effects, including immune modulatory activities. For example, some studies have documented superior therapeutic effects when resveratrol was given at low dietary-like concentrations than when administered at high concentrations (Cai et al., 2015) and although as mentioned before high doses of resveratrol are considered safe, available data come from short-term studies and thus the safety of this compound in long-term studies is unknown, and considering the fact that cancer chemopreventive studies are typically require several years of follow-up (Kotecha et al., 2016), the use of low doses of resveratrol may be logically a safest choice. Dealing with the poor bioavailability of resveratrol is another issue to consider and it is not surprising that several attempts have been made to develop sophisticated delivering system to enhance the resveratrol bioavailability or to identify resveratrol derivatives with better pharmacological profiles (Biasutto et al., 2017). Several resveratrol oligomers resulting from the polymerization of resveratrol monomer molecule to form dimers, trimmers, and more complex structures have been identified in nature and others have been artificially synthetized and many of them appear to have improved pharmacological profile in comparison with resveratrol monomer and this is an area of increasing scientific interest (Espinoza and Inaoka, 2017). In this regard, resveratrol derivatives from Melinjo extract (especially the resveratrol dimer Gnetin-C), an edible tree native of southeast Asia (Espinoza and Inaoka, 2017), have shown promising potential, including anticancer properties (Kato et al., 2009; Narayanan et al., 2015). Studies in humans have confirmed its safety and a superior bioavailability compared with its resveratrol monomer counterpart has been also documented (Tani et al., 2014).

Finally, the possibility of combining resveratrol with other immunotherapy agents is another attractive therapeutic approach that can be explored, either as a preventive or therapeutic intend, although well-designed preclinical studies are first required in order to define the efficacy and the safety of this approach.

## Author contributions

All authors listed have made a substantial, direct and intellectual contribution to the work, and approved it for publication.

### Conflict of interest statement

The authors declare that the research was conducted in the absence of any commercial or financial relationships that could be construed as a potential conflict of interest.
